# Racial disparities in Phase 1 COVID-19 vaccine shipments to Neighborhood sites in Pennsylvania by the Federal Retail Pharmacy Program

**DOI:** 10.1038/s41598-024-73116-1

**Published:** 2024-10-10

**Authors:** Geoffrey S. Holtzman, Yukun Yang, Pierce Louis, Stephen G. West, Piranavakumar Kandaswamy

**Affiliations:** 1 Philadelphia, PA USA; 2 Department of Communication Studies, Northeastern University, Boston, MA USA; 3 School of Public Health, Boston University, Boston, MA USA; 4 Department of Psychology, Arizona State University, Tempe, AZ USA; 5 Department of Computer Science, Boston University, Boston, MA USA

**Keywords:** Health policy, Public health, Medical ethics, Disease prevention, Health care economics, Vaccines

## Abstract

Early racial disparities in COVID-19 vaccination rates have been attributed primarily to personal vaccine attitudes and behavior. Little attention has been paid to the possibility that inequitable vaccine distribution may have contributed to racial disparities in vaccine uptake when supplies were most scarce. We test the hypothesis that scarce vaccines were distributed inequitably using the shipping addresses of 385,930 COVID-19 vaccine doses distributed in the first 17 weeks of Pennsylvania’s Phase 1 rollout (December 14, 2020 through April 12, 2021). All shipments we analyze were allocated via the Federal Retail Pharmacy Program, a public-private partnership coordinated by the Centers for Disease Control and Prevention.

Overall, White people had an average of 81.4% more retail pharmacy program doses shipped to their neighborhoods than did Black people. Regression models reveal that weekly vaccine allocations determined by pharmacy chains—rather than initial shipment and administration site decisions requiring state and federal approval—drove these effects. All findings remained consistent after controlling for neighborhood differences in income, population density, insurance coverage, number of pharmacies, and other social determinants of health.

Our findings suggest that the private distribution of scarce public resources should be assessed for racial impact, regulated as public resources, and monitored continuously.

**Supplementary Information:**

The online version contains supplementary material available at 10.1038/s41598-024-73116-1.

## Introduction

 Racial and ethnic disparities in COVID-19 vaccination rates in the U.S. have been widely discussed in the media and in scientific literature^1,2^. The dominant narrative has been that these disparities were driven by differences in vaccine hesitancy. This explanation is plausible, given the 400-year history of race-based medical inequities^3,4^. It is also supported by survey research, which has generally found greater COVID-19 vaccine hesitancy among Black people than White people in the U.S.^2,5–10^. However, there are both theoretical and empirical reasons to believe that this prevailing explanation for vaccination rate disparities in the U.S. is at best incomplete.

First, self-reported dispositions tend to be poor predictors of behavior in general^11^, and of COVID-19 vaccination in particular. In the first year, COVID-19 vaccines were available in the U.S., Black-White disparities in self-reported vaccine hesitancy in the U.S. declined only slightly^12,13^, but the corresponding difference in actual vaccine uptake dropped by over 90%^14–16^. Second, behavior depends not only on personal disposition but also on situational factors. In both the U.S. and U.K., Black people were more likely than White people to express vaccine hesitancy through February 2021, but only in the U.S. did Black people get vaccinated at lower rates^12^. These findings suggest that “issues related to access may underlie the observed lower vaccine uptake among minority populations in the U.S.”^12^ Approaches are needed to help understand and overcome such situational barriers to racial and ethnic equity in COVID-19 vaccine access in the U.S.

Linking neighborhood-level data on the availability of scarce resources (like new COVID-19 vaccines) to data on racial and ethnic demographics can facilitate such understanding. If demographic differences in health outcomes track geographic differences in scarce resource allocation, then racial and ethnic disparities in health outcomes may be reduced by more equitably allocating healthcare resources across neighborhoods. This approach has previously illuminated complex geodemographic relationships underlying Black-White disparities in heart disease (supermarket access^17^), cancer (air quality^18^), and other health outcomes^19^.

We draw on this approach to analyze the relationship between the racial and ethnic populations of 3,218 neighborhoods (defined in Census Bureau guidance as census tracts^20^) and the shipping destinations of COVID-19 vaccine doses in Pennsylvania. Because our emphasis is on the just distribution of scarce medical resources^21–24^, we focus on Phase 1 of Pennsylvania’s vaccine distribution plan (December 14, 2020 through April 12, 2021^25^). During this period, Pennsylvania prioritized vaccine eligibility for certain groups (e.g., all adults aged 65 or older) in an effort “to ensure ethical allocation of scarce vaccine,” according to the *PA COVID-19 Interim Vaccination Plan*^26^. Vaccines were administered to patients “free of charge”^27^ regardless of health insurance status, but providers were permitted and incentivized to bill private and federal medical insurance for each dose administered to patients with^26,28,29^ healthcare coverage.

Pennsylvania was the only U.S. state to provide separate datasets for doses allocated by its state Department of Health (DoH) and by corporate pharmacy chains enrolled in the Federal Retail Pharmacy Partnership (FRPP^30,31^). Distinguishing DoH from FRPP shipments was critical for our analyses, as the policies regulating the two programs—and thus the processes generating the data—differed in at least three important ways.

First, the degree of government oversight differed between programs. For DoH, state health department employees determined both shipping destination *site selection* and weekly *dose quantity* allocations^26,32^. FRPP *site selection* also required approval by state health department employees (as well as from the CDC^33,34^), but weekly FRPP dose allocations across retailers’ stores were determined by pharmacy corporations with little or no state or federal coordination or regulation^33,35–37^. According to a Government Accountability Office (GAO) Report to Congressional Committees, state health officials said they “had limited or no information about where vaccine doses were going or the amounts of doses allocated through CDC’s retail pharmacy program”^36^.

Second, ongoing DoH shipment decisions appear to have taken into consideration prior FRPP shipments^36^. Given the lack of information-sharing noted above, this may have created unique problems for DoH in assuring fair vaccine access for Pennsylvanians. State officials told the GAO that “the limited information on where vaccine doses were going made it difficult for states to optimally and equitably allocate and distribute their own limited supply of vaccine doses”^36^.

And third, DoH shipping addresses were not necessarily DoH vaccine administration sites. DoH vaccines were allocated to pharmacies, hospital systems, county health departments, and other public and private entities, many of which the state referred to as “depots” that had multiple points of service^26,38^. Reallocation to these points of service was not subject to further reporting requirements in Pennsylvania^26^. By contrast, recorded FRPP shipping addresses were, by policy, the final site of vaccine administration^26,35,29^. Given this lack of relevant information on the final sites of vaccine delivery through DoH, we limited our analysis to the 385,930 FRPP vaccine doses for which we were able to obtain shipping information.

With the data we obtained, we were able to test three hypotheses associated with three basic questions concerning vaccine equity:

### H1:

Were Whiter neighborhoods more likely to have at least one *site selected* to receive vaccine shipments?

### H2:

Among neighborhoods with at least one shipping destination site, did Whiter neighborhoods receive greater *dose quantities*?

### H3:

Did White people tend to have more total doses shipped to their neighborhoods overall?

Answering these questions may prove helpful in determining how to more equitably distribute, monitor, and regulate scarce medical resources during future public health emergencies^22^. To test the strength of our evidence and to explore potential causes of the hypothesized effects, we ran several models in addition to those conducted for our planned hypothesis tests^39,40^. We report these additional models briefly in the Results section, and describe them more fully in the supplementary material^41,42^.

## Results

### Descriptive statistics

Table 1 provides demographic information for all neighborhoods, as well as separate demographics for areas that did and did not have retail sites operated by pharmacy chains enrolled in FRPP.

**Table 1:** Demographics of neighborhoods that did and did not have retail sites operated by pharmacy chains enrolled in Pennsylvania’s FRPP.

	Neighborhoods without FRPP-eligible sites (*n* = 2,244)		Neighborhoods with FRPP-eligible sites (*n* = 974)		All neighborhoods in Pennsylvania (*n* = 3,218)		FRPP access by demographic category
**Race/ethnicity**							
White	6,686,434	(76.1%)	3,090,729	(77.1%)	9,777,163	(76.4%)	31.6%
Black	979,410	(11.2%)	383,633	(9.6%)	1,363,043	(10.7%)	28.1%
Hispanic or Latino	655,828	(7.5%)	299,388	(7.0%)	935,216	(7.3%)	32.0%
Other	460,557	(5.2%)	255,551	(6.4%)	716,108	(5.6%)	35.7%
**Age**							
<18	1,846,657	(21.0%)	815,734	(20.4%)	2,662,391	(20.8%)	30.1%
18–64	5,390,296	(61.4%)	2,457,123	(61.3%)	7,847,419	(61.3%)	31.3%
65+	1,545,276	(17.6%)	736,444	(18.4%)	2,281,720	(17.8%)	32.3%
**TOTALS**	8,492,920	(100%)	4,298,610	(100%)	12,791,530	(100%)	33.6%

FRPP-eligible sites are physical retail locations of pharmacy chains enrolled in Pennsylvania’s FRPP. FRPP access by demographic category is calculated as the percentage of all Pennsylvanians of a given race, ethnicity, or age living in neighborhoods with at least one FRPP-eligible pharmacy.

### Main hypothesis tests

#### H1:

Black population had a slightly stronger positive effect on the odds of a neighborhood receiving *any* FRPP vaccine sites than did White population. A neighborhood’s odds of having at least one FRPP vaccine shipment site increased by 13.7% for every 1,000 White residents (*OR* = 1.14) and by 21.2% (*OR* = 1.21) for every 1,000 Black residents (Table 2). Additional residents of other races or ethnicities had no significant incremental effect on either site selection or dose quantities.

**Table 2:** Effect of Black and White neighborhood populations on odds of receiving any vaccine.

Population	*B*	*SE*	95% CI		*p*	Exp(*B*)
			*LL*	*UL*		
**FRPP**						
Black	0.192	0.06	0.074	0.309	0.001	1.212
White	0.128	0.029	0.071	0.184	< .001	1.137

*B* = unstandardized regression coefficient; *SE* = standard error; CI = confidence interval. Exp(*B*) represents change in log odds of neighborhoods (*n* = 3,218) receiving any COVID-19 vaccine for every 1,000 residents of each race. Other races and Hispanic or Latino populations were included in initial logistic regression models but excluded by the model-fitting process.

#### H2:

Our regression models also showed that Black population had a significant negative effect on the quantity of doses shipped to such neighborhoods. Neighborhoods receiving vaccine received 191 fewer doses for every 1,000 Black residents (Table 3). Additional residents of other races or ethnicities had no significant incremental effect on either site selection or dose quantities.

**Table 3:** Expected additional doses per 1,000 neighborhood residents of each race.

Population	*R* ^2^	*F*	Estimate	*SE*	*p*	*t*
**FRPP** (*n* = 492)	0.039	19.70			< 0.001	
Intercept			874.9	44.2	< 0.001	19.784
Black			-190.9	43.0	< 0.001	4.438

*SE* = standard error. Sample sizes are determined by number of neighborhoods included in each program. Other races and Hispanic or Latino populations were included in initial linear regression models but excluded by the model-fitting process.

#### H3:

To calculate the net effects of these patterns on site selection and dose quantity on residents’ to FRPP vaccines in their neighborhoods, we ran a planned t-test of weighted mean doses. This test found that White people had an average of 81.4% more FRPP doses shipped to their neighborhoods (*M*_DOSES_ = 139.3) than Black people did (*M*_DOSES_ = 76.8), a difference that was statistically significant (*t* = 6.37, *p* < .001).

### Covariate models: adjusting for structural demographic factors

Models adjusted for neighborhood-level Social Vulnerability Index (SVI)^43^, median income^44^, population density, health insurance coverage rates^45^, and number of pharmacies^46^ are summarized below (Table 4) and reported in full in the supplementary material (Tables S1 and S2).

**Table 4:** Estimates with and without adjustments for median income, Social Vulnerability Index, population density, health insurance coverage, and number of eligible pharmacies.

Population	Odds ratio for receiving any vaccine		Expected additional doses of vaccine	
	Unadjusted	Adjusted	Unadjusted	Adjusted
**FRPP**				
Black	1.212	1.207	-190.9	-106.1
White	1.137	1.221	-	-

All estimates are calculated per 1,000 residents of each race. Full logistic regression models (for odds ratios) and linear regression models (for expected doses) are included in the supplementary material. Other races and Hispanic or Latino populations were included in the models but excluded by the model-fitting process. Exact p-values for unadjusted models are reported in Tables 2 and 3. Exact p-values for adjusted models are reported in Tables S2 and S3.

Odds of receiving any vaccine via the FRPP increased by 22.1% for every 1,000 White residents. (*OR* = 1.22) and 20.7% for every 1,000 Black residents (*OR* = 1.21). Neighborhoods receiving any vaccine received 106 fewer doses for every 1,000 Black residents, but additional residents of other races or ethnicities had no significant incremental effect on dose quantities.

### Alternative modeling techniques: sensitivity analyses

To probe the robustness of our findings, we used alternative approaches favored by researchers in some disciplines (e.g., epidemiology). The sign and statistical significance of all effects remained consistent with those in the corresponding regression models described above.

When we reassessed *site selection* via Poisson regressions with sandwich estimators (Tables S3 and S5), every 1,000 White residents increased the likelihood of a neighborhood receiving any FRPP vaccine by 11.2% (14.9% adjusted for covariates) and every 1,000 Black residents increased that likelihood by 17.2% (13.2% adjusted for covariates).

When we reassessed *dose quantities* via negative binomial regressions (Tables S4 and S6), every 1,000 Black residents was associated with 35.3% fewer FRPP doses (24.3% adjusted for covariates). Additional residents of other races or ethnicities had no significant incremental effect on dose quantities (Tables S4 and S6).

## Discussion

These findings support the view that Black-White racial disparities in COVID-19 vaccination rates during Phase 1 of Pennsylvania’s vaccination plan coincided with inequities in COVID-19 vaccine allocation^47^. Given that Black and White populations were the only significant population factors in our best-fitting regression models, we limit our discussion here to these two populations.

State-regulated FRPP site selection disproportionately favored neighborhoods with larger Black populations, but pharmacy corporations subsequently shipped far fewer FRPP doses to sites in neighborhoods with larger Black populations. This negative relationship was sufficiently large that, despite state regulation of initial site selection, White people had a statistically significant 81.4% more FRPP doses shipped to their neighborhoods than Black Pennsylvanians did.

These findings are consistent with a recent GAO report on the administration of vaccines allocated by retail corporations across the country^37^. According to that report, retailers enrolled in the FRPP did increase supply to socially vulnerable areas, but only “once vaccine supply became more readily available.”^48^ The report found that through at least September 2021, doses allocated by pharmacy chains enrolled in FRPP were less likely to be administered to Black Americans than were doses allocated by state health departments^48^.

However, neither we nor the GAO were able to analyze FRPP vaccine uptake data using actual doses administered before “April 2021, because this is when CDC began making data on COVID-19 vaccine doses distributed through the different federal vaccine distribution programs publicly available”^36^. Even for DoH, “race and ethnicity for COVID-19 vaccine recipients were missing for almost half (46.7%) of recipients,” with racial patterns of missingness that “may account for some, or even all, of the differences between shares of vaccinations and of the population by race and ethnicity”^48^. Thus, a major limitation of our study is that we have not been able to test whether vaccine allocation played any role in the fact that, “according to CDC”^48^, Federal Retail Pharmacy Program doses were disproportionately administered to White, non-Hispanic Americans through April 2021.

Our primary focus was to test for *prima facie* racially inequitable outcomes in COVID-19 allocation in a real-world context. However, our covariate models further suggest that the inequitable vaccine distribution we identified is not fully attributable to domain-general structural inequities in income, medical vulnerability, housing conditions^49^, health insurance coverage, or pharmacy location. Thus, federal policies guiding the private distribution of scarce vaccine may not only fail to counter well-known, domain-general structural inequities^33^, but may in fact exacerbate them.

We ran six additional models (see supplementary material) as sensitivity analyses to investigate whether the relationship between neighborhood demographics and vaccine shipments remained statistically significant after adjusting for the neighborhood characteristics of median income, population density, social vulnerability, insurance coverage, and number of pharmacies. None of these models weakens the evidence for racial inequities in COVID-19 vaccine allocation. Four of the models identified all the same increases in expected doses for neighborhoods with larger White populations and decrease in expected doses for neighborhoods with larger Black populations as did our initial models. And the two remaining models (Tables S1 and S5) indicated that any advantage in vaccine site selection conferred to neighborhoods with larger Black populations suggested in our initial models may be attributable to causes other than a concern for racial equity.

Our results concerning inequities in vaccine allocation may understate inequities in vaccine access (though not necessarily vaccine uptake)^47^. Black Pennsylvanians may have been less able to obtain vaccines outside their own neighborhoods, given racial disparities in transportation access^50^, work commute time^51^, and internet access^52^. Even for those living in neighborhoods with ample vaccine supply, we might expect racial inequities in the opportunity to be vaccinated, given that White people were up to 40% more likely than Black people to be eligible for telework during the COVID-19 pandemic^53–55^. These are critical issues for researchers, ethicists, supply chain experts, and policymakers to consider, but they can only be linked to actual vaccine uptake if demographic data on vaccine uptake are made available for future vaccine rollouts.

Even from a so-called “race-neutral” perspective^56^, where the goal is to reduce overall harm to society as a whole^57,58^, the racial disparities in vaccine allocation we have identified may have had significant disutility. The Pennsylvania COVID-19 Task Force allocation of initial doses was intended to reflect “disease epidemiology and local community factors”^26^. This suggests that greater dose quantities should be sent to neighborhoods where the risk of contracting and spreading COVID-19 was greatest. But compared to White workers, Black workers were not only less likely to work from home^53–55^, they were also 20% more likely to be frontline workers^59–61^ and three times more likely to take public transportation to work during the pandemic^50^. So why was *less* FRPP vaccine sent to areas with more Black people?

Medical ethicists and epidemiologists recognize that there can be tradeoffs between equity and overall utility^24,62^, but the allocation patterns we observed do not appear to have satisfied either virtue. Inequitable vaccine allocation may have increased risk of exposure to COVID-19 for everyone riding the bus^50^, working outside the home^53–55^, or buying food from grocery stores or restaurants^59–61^—potentially placing all people in those common spaces at risk of transmission and infection. Furthermore, by the end of Phase 1, approximately one in five doses allocated to Pennsylvania by the CDC had not been administered to anyone at all. Future research should therefore also examine whether inefficiencies in Pennsylvania’s vaccine rollout might have been remedied through more equitable distribution. Mathematical models for simultaneously improving both the equity and overall efficiency of the vaccine supply chain have long existed^63^, but calls to implement such approaches remain unheeded^64^.

It would be premature to conclude that the inequitable vaccine distribution patterns we discovered in these data were the result of racially targeted discrimination. Other differences between neighborhoods that correlate with race may have led corporations to allocate more vaccine to retail pharmacy locations whose patients were more likely to make in-store purchases—or to stores that were likely to have more customers. However, neither of these initially plausible explanations for the patterns of FRPP distribution we observed were supported by our adjusted models. To our surprise, pharmacy chains were more likely to have at least one FRPP site in neighborhoods with *lower* median incomes (Table S1)—and to ship greater dose quantities to sites in neighborhoods with *lower* population densities (Table S2).

To determine whether our findings about private vaccine allocations generalize across the country, additional analyses of other states’ data are needed. Such research, of course, depends on the availability of relevant data, but Pennsylvania may be the only state that publicly discloses its vaccine allocations. South Carolina, Louisiana, and Texas did so at one time, though none have since August 2021 and Texas’s data have been removed from the state’s website^65^. And even in Pennsylvania, policies permitting incomplete tracking of DoH shipments precluded comparison of these shipments with better-tracked FRPP shipments. Thus, while the Biden administration’s January 2021 national strategy emphasized data transparency as a key to equitable pandemic response, a lack of transparency remains an obstacle to achieving racial equity.

Sharing data on the allocation, distribution, and uptake of COVID-19 vaccines—as well as antivirals, biologics, and other supplies—is a critical part of narrowing the racial data gap^66^. Among other things, such data could benefit our colleagues who conduct research on vaccine hesitancy and with to control for extraneous variables that may affect the important relationship between vaccination intention and vaccine uptake. At present, a review of the COVID-19 inequities literature is marked by a glaring absence of empirical data on or conceptual consideration of vaccine allocation and distribution. This point is reinforced by the GAO report mentioned above, according to which more than a quarter of all retail program vaccinations reported by the CDC were missing race data as of January 9, 2022^48^.

Policies are urgently needed to increase oversight, transparency, and racial equity in the distribution of scarce resources during public health emergencies. Beginning in February 2021, the GAO made recommendations in at least five reports to Congress to address challenges like these^34,36,48,67,68^, but according to the U.S. Health and Human Services Office of the Inspector General, “all of GAO’s recommendations from these reports remain unimplemented” as of January 2023^38^. They are also unimplemented in the July 2023 Health and Human Services Commercialization Transition Guide^69^.

In addition to supporting the recommendations in the GAO reports, we make four recommendations based on our new analysis of the data:

1. Government programs that provide scarce medical resources to retail corporations for free or with appreciable government subsidy, but which allow them to profit by administering those resources, should be evaluated to assure that they maximize both efficient and equitable resource allocation.
2. Public health decisions that may be constrained or influenced by factors in the built environment should evaluated for their potential to amplify the effects of historical and contemporary inequities that may be embedded in that environment.
3. Oversight and communication regarding the *retail locations eligible* to receive scarce medical resources should, in general, be supported by continuous monitoring of and communication about the *actual*,* ongoing distribution* of those resources to their final sites of administration.
4. Tracking and sharing of racial and ethnic data on the *actual*,* ongoing uptake* of scarce medical resources *by members of the public* should be initiated and maintained for the duration of public-private partnerships, with enforcement of minimal standards of data completeness.

The potential consequences of ignoring or overlooking these considerations are perhaps best illustrated in a GAO report to Congressional Committees from November 2021:

[P]harmacy partners in one state chose to distribute vaccine doses to pharmacy sites the state had not identified as priorities because they were located in areas with a sufficient number of existing vaccine providers. Health officials in the state said they had to negotiate with pharmacy partners participating in CDC’s retail pharmacy program to send vaccine doses to pharmacy sites in higher-need areas of their state^36^.

We urge the research community to engage in a deeper analysis of potential inequities in the physical distribution of scarce medical resources before the next public health emergency. We also urge policymakers to take these issues more seriously. According to CDC data, over 250,000 Americans died of COVID-19 during the 17-week period studied here^70^.

## Methods

### Study Design

During the week of Martin Luther King Jr. Day (January 17, 2021), the Federal Retail Partnership Program (FRPP) began allocating and distributing Moderna vaccine (minimum lot size: 100 doses) to Rite Aid Corporation and Topco Associates LLC, which subsequently shipped those vaccines to retail pharmacy locations of their choosing. Starting the week of February 21, Rite Aid Corporation and Topco Associates LLC—as well as CVS Pharmacy Inc., and Walmart Inc.— began receiving Pfizer vaccine (minimum lot size: 450 doses) via the FRPP and shipping those doses to their respective retail locations. After the week of March 8, 2021, FRPP shipments in Pennsylvania ceased to be reported on the DoH website. Phase 1 vaccinations in Pennsylvania concluded on April 12, 2021^25^.

All available data concerning the quantities and destinations of COVID-19 vaccine shipped to Pennsylvania were downloaded from the Department of Health website^25^, to which they were posted by the state on a weekly basis^25,26^. Analytic decisions regarding how to distinguish the processes in our models were made on the basis of U.S. Government Accountability Office Reports to Congress (GAO-21-443^34^, GAO-22-104457^36^, GAO-22-105079^48^) and the Pennsylvania COVID-19 Interim Vaccination Plan^26,32,29,33^.

Our primary outcome measures were site selection (a dichotomous variable); and shipment quantity (a count variable), conditional on the presence of at least one eligible destination site. Our predictors were racial and ethnic population subtotals within neighborhoods, defined here as census tracts. According to the Census Bureau, “Census tracts are designed to follow the boundaries of neighborhoods”^20^, and they are reported to be the geographic level at which the PA DoH identified vulnerable populations for their vaccination plan^26^.

Estimates of the racial and ethnic population totals in each of Pennsylvania’s 3,218 census tracts were obtained from table DP05 of the 2015–2019 American Community Survey (ACS) 5-Year Estimate Data Profiles^71^. Since geodemographic data in the U.S. reflect historical and current practices of hypodescent (“the one-drop rule”^72^), we drew on estimates of populations identifying as “One race, [Race], not Hispanic or Latino,” “Multiracial, not Hispanic or Latino,” and “Hispanic or Latino.” In all cases, we use the verbatim racial and ethnic terminology associated with the census data.

After all hypothesis tests on the data just described were completed, Census tract-level data on median income^44^, Social Vulnerability Index (SVI)^43^, population density^43^, health insurance coverage rates^45^, and number of FRPP-eligible pharmacies^46^ were joined to them to probe the robustness of our *prima facie* findings to demographic indicators potentially reflecting structural racism.

### Data processing

After resolving formatting inconsistencies across public datasets and between those datasets and USPS address standards, we identified each vaccine shipment with the census tract of its destination. To achieve this, we uploaded all shipment addresses to the Census Bureau’s Geocoding Services Web Application Interface (API) for batched geographic identification. Addresses that were not initially geoidentified successfully were then mapped to their geographic coordinates (geocoded) via the Google Maps API. These coordinates were then uploaded to the Census Bureau’s geocoding API for batched geographic identification.

All addresses that remained unidentified due to absent or errant city or ZIP code data (0.28%) were manually assigned to the PA census tracts of identical street addresses elsewhere in our dataset. Missing dose quantities for individual shipments were small (0.39%) and limited to files we obtained for the first and fourth weeks of FRPP. In those files, *all* observed shipment quantities were 100 doses—with no variation. Little and Rubin state that in such an instance—where there is no variation in observed values—the most appropriate method of imputation may be to replace missing cells with the “constant value”^73^. We therefore imputed all missing dose quantities as 100 doses^74^.

### Hypothesis tests

The allocation of any vaccine to a neighborhood depends on the prior availability of suitable vaccination sites and cold storage facilities. In contrast, the quantity of doses shipped to neighborhoods in which these sites and facilities are available is somewhat more discretionary. In other words, the processes leading to these two outcomes are distinct. As described above, the entities and policies determining *site selection* were distinct from those determining *dose quantities*. We therefore ran three analyses for each dataset.

For each dataset, we first ran logistic regressions to estimate the effects of racial and ethnic population subtotals on receipt of any vaccine. Logistic regression predominates in the analysis of non-rare binary outcomes for several reasons, including the “advantages of interpretation […] in terms of the odds”^75^ of an outcome and the ability to support “strong inferences about the magnitude of effects”^75^. To reduce the risk of confirmation bias, variable selection for all final logistic regression models followed the purposeful selection procedure described by Hosmer and Lemeshow^76^ This method, which includes measures to avoid both model overfitting and the premature exclusion of potentially important variables, is more labor-intensive but also better-performing than traditional automated subset selection methods such as backward and stepwise selection^77^.

Second, we conducted linear regressions to estimate expected dose quantities for neighborhoods in each allocation program as a function of racial and ethnic population subtotals. Corresponding to the null hypothesis that persons of all races should be equally important in vaccine site selection and allocation, best-fitting models in the main text were selected using the approach described in Cohen et al., whereby “terms are removed sequentially as they fail to contribute to *R*^2^”^75^.

Third, we estimated the net impact of site selection and dose quantity effects on Black and White populations’ relative opportunity to be vaccinated. For these estimates, we report the average number of doses to which Black and White Pennsylvanians had neighborhood-level access. In line with existing research into geodemographic health inequities^78,79^, we calculated these averages as weighted mean doses for racial subpopulations across all neighborhoods in our dataset:

$$\frac {\sum\limits_{\text{tract} = 1}^{3218} {\text{subpopulation}}_{\text{tract}} * {\text{doses}}_{\text{tract}}} {\sum\limits_{\text{tract} = 1}^{3218} {\text{subpopulation}}_{\text{tract}}}$$

Weighted variance was calculated with this same approach to provide standard deviations and facilitate reporting of population-weighted t-tests^80^. All statistical tests were two-tailed. Details for all covariate models and alternative modeling techniques are provided in supplementary material.

### Tests of robustness of findings: sensitivity analyses with and without covariates

We briefly describe results from the sensitivity analyses in the Results section of the main text, and provide further details on their rationales^49,81–85^, methods^43−46,86–94^, results, and interpretations in the supplementary material.

## Electronic supplementary material

Below is the link to the electronic supplementary material.

Supplementary Material 1

## Data Availability

All data and code needed to reproduce the analyses described herein are publicly available at https://osf.io/yx6vd/?view_only=47b6411891f64cd1ac6711e92440f39e.
