# Supplementary material for: Racial disparities in Phase 1 COVID-19 vaccine shipments to Neighborhood sites in Pennsylvania by the Federal Retail Pharmacy Program
Source: Sci Rep. 2024 Oct 10;14:23591. doi: 10.1038/s41598-024-73116-1 (PMC11466953; doi:10.1038/s41598-024-73116-1)
Supplement: Supplementary file 1 — Supplementary Material 1 [file 41598_2024_73116_MOESM1_ESM.pdf]

## Supplemental materials

### Covariate models

Our principal interest in the main text was to determine whether and at what stage there were racial inequities in COVID-19 vaccine distribution. However, readers of earlier drafts expressed strong interest in seeing exploratory models that might shed light on the underlying causes of the effects we discovered and address potential confounders. We received suggestions to include over forty potential covariates, but for statistical[34,35], epistemological[36,37], and practical reasons, we limited data collection and analysis to five covariates:

- Median income, which is one of the most commonly used socioeconomic control variables in public health research and is most likely to be associated with FRPP shipment quantities.

- Population density, which is determined by population and land area, and which is also widely used to estimate urbanicity.

- Social Vulnerability Index (SVI), a CDC metric that ranks census tracts on 15 American Community Survey (ACS) factors "to help public officials and local planners better prepare communities to respond to emergency events."

- Insurance rate, calculated as persons with health insurance coverage per one hundred residents.

- Pharmacy count, defined as the total number of pharmacy locations eligible for enrollment as Pennsylvania FRPP sites

We included median income for theoretical reasons discussed in the main text, and included SVI based on its inclusion in policy documents described there as well. We included population density to test the objection that racial disparities in site selection could be justified on the grounds that tightly packed census tracts in urban areas (which tend to relatively greater Blacker) did not need as many sites as census tracts in sprawling rural areas (which also tend to be Blacker). Percent of residents with health insurance was included because vaccine administration was free to recipients, but pharmacies were permitted to bill insurance companies for each dose administered to a covered individual[5,27,30]. The number of FRPP-approved pharmacies was included to see whether racial effects attributed in our initial models to inequitable vaccine distribution could be attributed to racial differences in neighborhood pharmacy presence.

Data on median income, Social Vulnerability Index (SVI), population density, health insurance coverage, and number of eligible pharmacies were downloaded and joined to vaccine shipment data at the Census tract level after completion of all primary analyses presented in the main text. Median income and health insurance coverage data were obtained from tables S1903 and

S27015, respectively, of the 2015-2019 American Community Survey 5-Year Estimate Data Profiles[43,39]. Census tract-level SVI and population density were obtained from the CDC's Agency for Toxic Substances and Disease Registry data for 2018[44] Locations of all pharmacies in Pennsylvania were obtained from Pennsylvania's Pharmacy Locator website.

To facilitate the interpretation of odds ratios, we followed a convention in our supplemental tables of reporting effects of pharmacy count and SVI (which can range from 0 to 1) for every change of 0.1, effects of population density for every 1,000 people per square mile, and effects of median income per $10,000. We report the effects of percent health insurance coverage unscaled. These scaling decisions provide more interpretable regression coefficients, but do not affect tests of statistical significance. In order to estimate the effects of racial subpopulations over and above effects that might be explained by these covariates, all covariates were entered into these models first at each stage in the model-fitting process[47]. For all covariate models (Tables S1, S2, S5, and S6), census tracts for which covariate data were unavailable were excluded listwise.

*Site selection*

The receipt of any FRPP vaccine was associated with higher pharmacy counts and with lower median income (Table S1). Even after adjustments, the sign and significance of our unadjusted effects of race on site selection remained unchanged from those in our unadjusted model (Table 2 in main article).

**Table S1—Adjusted logistic regression models for site selection**

| Allocation program | *B* | *SE* | 95% CI | | *p* | Exp(*B*) |
|---|---|---|---|---|---|---|
| | | | *LL* | *UL* | | |
| **FRPP** | | | | | | |
| SVI | 0.024 | 0.033 | -0.040 | 0.089 | .461 | 1.025 |
| Income | -0.136 | 0.035 | -0.204 | -0.067 | < .001 | 0.873 |
| Density | 0.009 | 0.009 | -0.008 | 0.026 | .301 | 1.009 |
| Insurance | 0.024 | 0.015 | -0.006 | 0.053 | .120 | 1.024 |
| Pharmacies | 0.129 | 0.007 | 0.116 | 0.142 | < .001 | 1.138 |
| White | 0.199 | 0.039 | 0.123 | 0.276 | < .001 | 1.221 |
| Black | 0.188 | 0.076 | 0.040 | 0.337 | .013 | 1.207 |

*Note*. *Note*. *B* = unstandardized regression coefficient; *SE* = standard error; CI = confidence interval. Exp(*B*) estimates the change in log odds of neighborhoods (*n* = 3,179) receiving any COVID-19 vaccine for every 1,000 residents of each race after adjustment for SVI, median income, population density, health insurance coverage, and the number of pharmacies. Other races and Hispanic or Latino populations were included in initial logistic regression models but excluded by the model-fitting process.

*Dose quantities*

FRPP sent fewer doses to more densely-populated areas than to areas of lesser population density (Table S2). Even after adjustments, the sign and significance of our unadjusted effects of race on dose quantities remained unchanged from those in our unadjusted model (Table 3).

**Table S2—Adjusted linear regression models for dose quantities**

| Allocation program | $R^2$ | $F$ | Estimate | $SE$ | $p$ |
|---|---|---|---|---|---|
| **FRPP** ($n$ = 488) | .071 | 6.167 | | | < .001 |
| Intercept | | | 234.2 | 1053.3 | .824 |
| SVI | | | -1.112 | 21.5 | .958 |
| Income | | | -18.1 | 22.0 | .410 |
| Density | | | -19.8 | 5.6 | < .001 |
| Insurance | | | 8.5 | 10.4 | .415 |
| Pharmacies | | | 0.566 | 3.652 | .877 |
| Black | | | -106.1 | 48.2 | .028 |

*Note*. *SE* = standard error. Estimates reflect expected additional doses per 1,000 neighborhood residents of each race. Other races and White, Hispanic or Latino populations were included in initial logistic regression models but excluded by the model-fitting process.

## Alternative modeling techniques

*Site selection*

Our shipping site selection models examined the receipt of any vaccine, a desirable outcome that would not typically be characterized as a *risk* or a *prevalence*. For this reason, we reported our findings concerning the absence or presence of any shipping destination sites in neighborhoods in terms of odds ratios, calculated via logistic regressions, in the main text. Logistic regression predominates in the analysis of non-rare binary outcomes for several reasons, including the "advantages of interpretation of regression coefficients in terms of the *odds*"[47] of an outcome and the ability to support "strong inferences about the magnitude of effects"[47].

Though the use of logistic regressions to calculate effect sizes for dichotomous outcomes is common across the behavioral sciences, in epidemiology it has become increasingly common to conduct Poisson regression with sandwich estimators[56,60]. Poisson regression has been used by epidemiologists to report risk ratios[61] and relative risk[62,63] in clinical trials and cohort studies, as well as the prevalence ratio in cross-sectional studies[60] (sometimes mistakenly referred to as "prevalence rate ratio"[64]).

For this reason, some readers may prefer or be more familiar with risk ratios[55]—calculated via Poisson regression with sandwich estimators—rather than odds ratios calculated via logistic

regression. We, therefore, report these as well in our supplemental tables. We also report the exponentiation of the race variables in all equations, which we interpret and report in the main text. This is how risk ratios are calculated from Poisson regressions, and in our case, they represent relative probabilities of receiving at least some vaccine shipments.

The sign, significance, and order of all effects in these Poisson regressions (Table S3) were consistent in all cases with those in our logistic regressions (Table 2). All effect sizes derived from Poisson regressions in *prima facie* analyses were between 2.2% and 3.3% smaller than those derived from logistic regressions.

**Table S3—Poisson regressions with sandwich estimators for likelihood of receiving any vaccine**

| Allocation program | *B* | *SE* | 95% CI | | *p* | Exp(*B*) |
|---|---|---|---|---|---|---|
| | | | *LL* | *UL* | | |
| **FRPP** | | | | | | |
| Black | 0.159 | 0.048 | 0.065 | 0.252 | < .001 | 1.172 |
| White | 0.107 | 0.023 | 0.060 | 0.153 | < .001 | 1.112 |

*Note*. *B* = unstandardized regression coefficient; *SE* = standard error; CI = confidence interval. Exp(*B*) estimates the change in risk ratio of neighborhoods (n = 3,179) receiving any COVID-19 vaccine for every 1,000 residents of each race. Other races and Hispanic or Latino populations were included in initial logistic regression models but excluded by the model-fitting process.

*Dose quantities*

A different set of methodological considerations arises for dose shipment quantities. Quantities of vaccine doses shipped to neighborhoods are not, strictly speaking, a continuous outcome, as COVID-19 vaccine vials contain multiple doses and all dose quantities in our data are (appropriately) recorded as integers. At the same time, these quantities do not represent the typical characterization in Poisson models as "the number of occurrences of an event in a fixed period of time"[59]. Further, the government documents described in the main text make clear that each of the events contributing to the count is not "independent"[59], an assumption of Poisson regression.

In the main text, we follow the common (and easy-to-interpret) convention of modeling shipment quantities via linear regressions with measured variables. These analyses are theoretically expected to approximate the Poisson results except when the mean predicted count is low (which was not the case for our data). However, in consideration of the perspective that these quantities might appropriately be treated as counts, we reanalyzed shipment dose quantities in this supplement as counts using negative binomial regressions, which are less sensitive to violations of assumptions than basic Poisson regression[67,68].

The sign and significance of all effects in our negative binomial regression results (Table S4) were consistent in all cases with those in our corresponding linear regressions (Table 3). The exponentiation of the regression coefficient represents the multiplicative effect of 1,000 residents of each race on the expected number of doses received. Every 1,000 Black residents

was associated with 35.3% fewer FRPP doses, and adding white residents to the model did not improve model fit.

**Table S4—Negative binomial regressions for expected additional doses.**

| Allocation program | Estimate | *SE* | *p* | Exp(*B*) |
|---|---|---|---|---|
| **FRPP** (*n* = 492) | | | | |
| Intercept | 6.817 | 0.043 | < .001 | |
| Black | -0.435 | 0.042 | < .001 | 0.647 |

*Note*. *SE* = standard error. Exp(*B*) reflects the expected proportional change in doses per 1,000 residents of each race. Sample sizes are determined by number of neighborhoods included in each program. Other races, White, and Hispanic or Latino populations were included in initial linear regression models but excluded by the model-fitting process.

## Alternative covariate models

All coefficients in all covariate models using Poisson or negative binomial regression were of the same sign, significance, and relative magnitude as those in the corresponding logistic and linear regression models with covariates (Tables S1 and S2).

### *Site selection*

**Table S5—Adjusted Poisson regression models with sandwich estimators.**

| Allocation program | *B* | *SE* | 95% CI | | *p* | Exp(*B*) |
|---|---|---|---|---|---|---|
| | | | *LL* | *UL* | | |
| **FRPP** | | | | | | |
| SVI | 0.022 | 0.023 | -0.032 | 0.075 | .338 | 1.022 |
| Income | -0.098 | 0.029 | -0.155 | -0.041 | < .001 | 0.907 |
| Density | 0.008 | 0.007 | -0.007 | 0.022 | .260 | 1.008 |
| Insurance | 0.021 | 0.011 | -0.004 | 0.047 | .061 | 1.235 |
| Pharmacies | 0.069 | 0.037 | 0.063 | 0.076 | < .001 | 1.072 |
| White | 0.139 | 0.029 | 0.078 | 0.201 | < .001 | 1.149 |
| Black | 0.124 | 0.060 | 0.001 | 0.249 | .042 | 1.132 |

*Note*. *B* = unstandardized regression coefficient; *SE* = standard error; CI = confidence interval. Exp(*B*) estimates the change in risk ratio of neighborhoods (*n* = 3,179) receiving any COVID-19 vaccine for every 1,000 residents of each race after adjustment for SVI, median income, population density, health insurance coverage, and the number of pharmacies. Other races and Hispanic or Latino populations were included in initial logistic regression models but excluded by the model-fitting process.

*Dose quantities*

**Table S6—Adjusted negative binomial regressions for expected additional doses.**

| Allocation program | Estimate | *SE* | *p* | Exp(*B*) |
|---|---|---|---|---|
| **FRPP** (*n* = 492) | | | | |
| Intercept | 6.237 | 1.058 | < .001 | |
| SVI | 0.007 | 0.022 | .734 | 1.007 |
| Income | -0.025 | 0.022 | .264 | 0.976 |
| Density | -0.045 | 0.006 | < .001 | 0.956 |
| Insurance | 0.009 | 0.010 | .412 | 1.009 |
| Pharmacies | -0.004 | 0.004 | .914 | 1.000 |
| Black | -0.278 | 0.049 | < .001 | 0.757 |

*Note*. *SE* = standard error. Exp(*B*) reflects the expected proportional change in doses per 1,000 residents of each race after adjustment for SVI, median income, population density, health insurance coverage, and the number of pharmacies.. Sample sizes are determined by the number of neighborhoods included in each program. Other races, White, and Hispanic or Latino populations were included in initial linear regression models but excluded by the model-fitting process.

## FRPP distribution by the manufacturer

*Site selection*

None of the government documents we obtained and reviewed mentioned any differences in the distribution of the Moderna and Pfizer vaccines for either the FRPP or DoH programs, though differences in dosing schedule, approved age range, and storage requirements are provided in the appendix on FDA authorization of the vaccines in one GAO report[91]. We therefore analyzed FRPP shipments as a single dataset, in line with conventions, in the main text. Here, we provide separate analyses of Moderna FRPP and Pfizer FRPP vaccines for exploratory purposes.

Larger White populations were associated with greater odds of receiving at least some Pfizer vaccines through the FRPP (Table S7). Larger White populations and larger Black populations were associated with greater odds of receiving at least some Moderna vaccines through the FRPP.

**Table S7—Logistic regression models for site selection by FRPP manufacturer**

| Allocation program | *B* | *SE* | 95% CI | | *p* | Exp(*B*) |
|---|---|---|---|---|---|---|
| | | | *LL* | *UL* | | |
| **Pfizer** (*n* = 49) | | | | | | |
| White | .150 | .075 | 0.003 | 0.297 | .045 | 1.162 |
| **Moderna** (*n* = 458) | | | | | | |
| Black | .219 | .060 | 0.101 | 0.337 | < .001 | 1.245 |
| White | .136 | .030 | 0.078 | 0.194 | < .001 | 1.146 |

*Note*. *Note*. *B* = unstandardized regression coefficient; *SE* = standard error; CI = confidence interval. Exp(*B*) estimates the change in log odds of neighborhoods (*n* = 3,179) receiving any COVID-19 vaccine for every 1,000 residents of each race after adjustment for SVI, median income, and population density. Other races, Black, and Hispanic or Latino populations were included in initial logistic regression model for Pfizer, but excluded by the model-fitting process. Other races and Hispanic or Latino populations were included in initial logistic regression model for Pfizer, but excluded by the model-fitting process.

*Dose quantities*

Neighborhoods with larger Black populations receiving shipments tended to receive fewer doses of Moderna FRPP vaccines (Table S8).

**Table S8—Linear regression models for dose quantities by FRPP manufacturer**

| Allocation program | $R^2$ | *F* | Estimate | *SE* | *p* |
|---|---|---|---|---|---|
| **Pfizer** (*n* = 49) | .055 | 2.72 | | | .106 |
| Intercept | | | 2897.59 | 263.45 | < .001 |
| Black | | | -1218.95 | 739.54 | .106 |
| **Moderna** (*n* = 458) | .104 | 26.46 | | | < .001 |
| Intercept | | | 633.96 | 19.05 | < .001 |
| Black | | | -118.68 | 17.41 | < .001 |
| All other races | | | -1656.33 | 694.02 | .017 |

*Note*. *SE* = standard error. Estimates reflect expected additional doses per 1,000 neighborhood residents of each race. Other races, White, and Hispanic or Latino populations were included in initial logistic regression models but excluded by the model-fitting process.

Note that the Pfizer-FRPP model in table S8 is at conventionally unacceptable risk of both Type I error ($p$ = .106) error and Type II error (achieved power $1 - \beta = .378$)[35]. The results of this model would traditionally be characterized as inconclusive, but we include it in Table S8 for completeness.
